# Aberration Estimation for Synthetic Aperture Digital Holographic Microscope Using Deep Neural Network

**DOI:** 10.3390/s23229278

**Published:** 2023-11-20

**Authors:** Hosung Jeon, Minwoo Jung, Gunhee Lee, Joonku Hahn

**Affiliations:** School of Electronic and Electrical Engineering, Kyungpook National University, Daegu 41566, Republic of Korea; jhs0485@knu.ac.kr (H.J.); apvmf12@knu.ac.kr (M.J.); gunhee7765@knu.ac.kr (G.L.)

**Keywords:** synthetic aperture, holographic microscope, deep neural network, aberration estimation

## Abstract

Digital holographic microscopy (DHM) is a valuable technique for investigating the optical properties of samples through the measurement of intensity and phase of diffracted beams. However, DHMs are constrained by Lagrange invariance, compromising the spatial bandwidth product (SBP) which relates resolution and field of view. Synthetic aperture DHM (SA-DHM) was introduced to overcome this limitation, but it faces significant challenges such as aberrations in synthesizing the optical information corresponding to the steering angle of incident wave. This paper proposes a novel approach utilizing deep neural networks (DNNs) for compensating aberrations in SA-DHM, extending the compensation scope beyond the numerical aperture (NA) of the objective lens. The method involves training a DNN from diffraction patterns and Zernike coefficients through a circular aperture, enabling effective aberration compensation in the illumination beam. This method makes it possible to estimate aberration coefficients from the only part of the diffracted beam cutoff by the circular aperture mask. With the proposed technique, the simulation results present improved resolution and quality of sample images. The integration of deep neural networks with SA-DHM holds promise for advancing microscopy capabilities and overcoming existing limitations.

## 1. Introduction

Digital holographic microscopy (DHM) is a technique for obtaining optical information of a sample by interfering the diffracted beam from the sample to be measured with a reference beam [1,2,3,4]. The great advantage of DHM is to understand the optical properties of a sample by measuring the intensity and phase of the diffracted beam from the sample. In addition, defects such as aberrations in optical elements or dust can be distinguished in the measured wavefront and the errors can be removed from the signal field to obtain more accurate information about the sample. Due to their many advantages, DHMs have been used in a variety of fields, including biological, microcircuit, and polymer characterization. However, DHMs also suffer from the same limitations as conventional optical microscopy, known as Lagrange invariance. In DHMs, Lagrange invariance represents the spatial bandwidth product (SBP) [5,6], which is the product of resolution and field of view, with the trade-off that higher resolution results in a smaller field of view.

The synthetic aperture DHM (SA-DHM) is designed to overcome the SBP limit [7,8,9,10]. SA-DHMs have a special method to illuminate a sample. It is the control of the direction of the reference beam [11,12,13], which can be realized by using either a Galvano mirror or SLM together with a condenser lens. The measured optical information of the sample is shifted in the Fourier domain depending on the spatial frequency of the reference beam, and it is synthesized for each direction of the reference beam. The synthesized optical information can theoretically be higher than the spatial resolution of the laser used to record the hologram. However, when the steering angle of the reference beam is increased to achieve higher resolution, the quality of the illumination beam decreases due to the change of states of some optics, and it causes aberrations of the illumination beam. Therefore, the resolution of the synthesized optical information generally decreases.

The aberrations of this reference beam must be removed from the measured optical information of the sample prior to synthesis. The simplest way is to directly measure the phase profile of the reference beam and subtract the reference phase profile from the acquired sample phase [14,15,16,17]. Since the synthetic aperture requires so many data acquisitions, it is very difficult to measure each phase profile of the reference beam. To solve this problem, an iterative algorithm is used [15,18,19]. The iterative algorithm separates the signal and aberration regions and extracts the Zernike polynomial coefficients of the aberration. The extracted Zernike coefficients are then numerically restored to the phase profile of the aberration and subtracted from the phase of the measured sample. However, the iterative algorithm takes a long time to compute the coefficients, and the main problem is that a reference beam with an angle larger than the numerical aperture (NA) of the objective lens makes it very difficult to completely remove the aberration because the beam is not observed by the camera of the DHM.

In recent years, the deep neural network framework has been rapidly developed and has made great contributions to the field of microscopy [20,21,22]. In particular, a proposition was made to analyze the aberration by extracting the Zernike coefficient using ResNet from the measured phase profile, and the aberration compensation using ResNet shows excellent results [23,24]. However, this method also needs to measure the aberration of the reference beam, which means that it cannot compensate for aberrations larger than the NA of the objective lens. In this paper, we propose a method to compensate aberrations in the reference beam not only within but also outside the NA of the objective lens using a deep neural network. In this paper, we propose a method to train a neural network using diffraction pattern and Zernike coefficients from a circular aperture defining the Zernike domain, and to compensate the aberration of the reference beam based. Some additional constraints in the aperture such as a coded aperture may be helpful to estimate the aberration functions, but we intend to use the circular aperture by virtue of having no need to modify the optics and a benefit of the efficiency of the optical power. Our proposed method effectively compensates for the aberrations of the reference beam, and simulation results show that high-resolution sample images can be obtained from SA-DHM.

## 2. Aberration of Synthetic Aperture Digital Holographic Microscope

**Figure 1:**
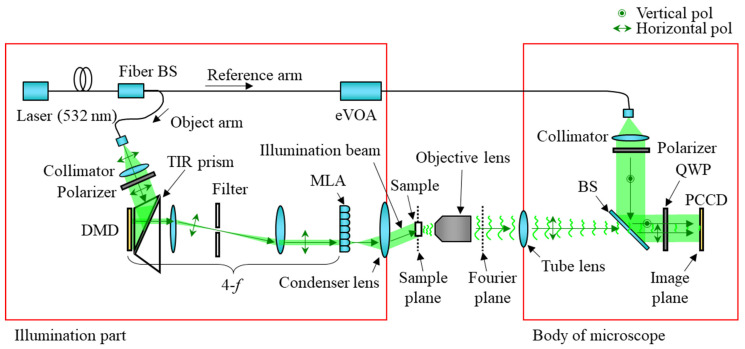
The structure of a synthetic aperture digital holographic microscope.

SA-DHM has a similar structure to a digital holographic microscope, consisting of an objective lens and a tube lens that are a 4-*f* optical system, including a sample plane and an image plane, and it has additional optics, which is illumination optics of the reference beam to interfere an object beam and a reference beam. SA-DHM has a special optical system that manipulates the direction of the illumination beam to measure the high-frequency term of the signal of a sample. Figure 1 shows our SA-DHM. In our system, the pig-tailed laser is used with 532 nm. The attached fiber is polarization-maintaining single mode optical fiber; hence, some optics that increase spatial coherence of the beam are not necessary and this helps keep our system compact. The laser beam is guided to the fiber beam splitter, and it is separated into the object arm and the reference arm. In the object arm, the laser beam is collimated by the collimator after releasing from the optical fiber, and it modulated by the digital micro-mirror device (DMD). It is noteworthy that the DMD is later responsible for determining the direction of the sample illumination. The modulated beam by the DMD is transmitted to the micro-lens array (MLA) after being magnified by two times at the same time as the high-order term is removed by the filter in the 4-*f* system. When the DMD displays a pattern to illuminate one lens on the MLA, the lens condenses the beam at one point, and the beam becomes a point source. This point source becomes a directional plane wave and illuminates the sample. After illuminating the sample by the illumination beam, the beam is diffracted by the sample, then the diffracted beam is transmitted to the polarized charge-coupled device (PCCD) by the object lens and tube lens. The reference beam in the reference arm is directly radiated to PCCD to generate interference pattern with the object beam. It is noteworthy that the polarizer and quarter-wave plate are used before the PCCD. They generate 4-step phase shifted interference patterns on the PCCD [25].

**Figure 2:**
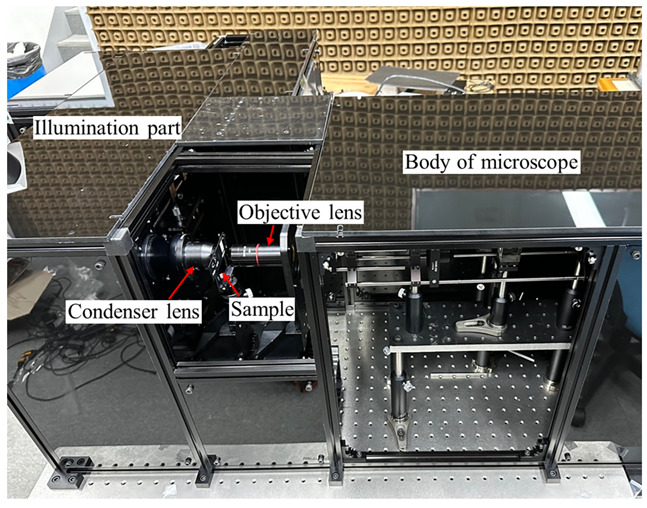
Implemented synthetic aperture digital holographic microscope.

Figure 2 shows our SA-DHM. A fiber beam splitter is used to distribute the beam in an optical power ratio of 99% for the object arm and 1% for the reference arm. The eVOA of Thorlabs on the reference arm controls the optical power from 0 to 5 V. The DMD has a resolution of 2560 × 1600 and a pixel pitch of 7.6 μm from Vialux. The 4-*f* optics consist of 2-inch diameter lenses with focal lengths of 100 mm and 150 mm, respectively. The MLA with a focal length of 3.26 mm and a lens pitch of 1.3 mm is made by Okotech. The condenser lens is an achromatic lens with an NA of 0.78, a clear aperture of 30 mm, and a WD of 6.6 mm. We used a 4× objective with an NA of 0.2 and a WD of 20 mm from Navitar and a 1× tube lens with a focal length of 200 mm from Thorlabs. The PCCD is an FLIR polarization monochromatic camera with a resolution of 2448 × 2048 and a pixel pitch of 3.45 um. A 1-inch QWP is placed directly in front of the PCCD to implement a parallel phase-shifting DHM [25].

When measuring the sample, the beam from the MLA is collimated by the condenser lens, and the beam has a designated direction. Here, the wave vector $\vec{k_{i}}$ of the directional plane wave from the optical axis is calculated by

(1) $$\vec{k_{i}}=\frac{k}{\sqrt{{\left(Mx_{i}\right)}^{2}+{\left(My_{i}\right)}^{2}+{\left(WD_{condenser}\right)}^{2}}}\left(Mx_{i},My_{i},WD_{condenser}\right)$$

where *M* is the magnification factor of the 4-*f* system; *p* and *q* are the index number of a lens of the MLA. A sample diffracts the illumination beam from these angles, and the diffracted beam is imaged on the PCCD.

In the reference arm, the reference beam divided by the fiber beam splitter passes through the eVOA, which controls the optical power of the passing beam depending on the input voltage. The reason for using the eVOA is to match the total optical power to the object beam so that the contrast of an interference pattern on the PCCD increases, thereby increasing the signal-to-noise ratio. The beam from the optical fiber is also collimated by the collimator, and it is directly heading to the PCCD. Then, the object beam and the reference beam are interfered on the PCCD, so they form a hologram. Since the PCCD has 4-direction linear polarizers for each of 4 pixels, 4-phase shifted holograms can be acquired at once. The holograms are expressed as follows:

(2) $$I_{n}={\left|A_{o}\right|}^{2}+{\left|A_{r}\right|}^{2}+2A_{o}A_{r}\cos\left(\theta_{o}+\frac{n\pi }{2}\right),$$

where $I_{n}$ are 4-phase shifted hologram patterns captured by the PCCD, *A_o_* and *A_r_* are amplitude of the object and reference beam, and $\theta_{o}$ is the phase profile of the object beam. Using Equation (2), we can numerically reconstruct the complex wave function $U_{O}$ of the object, and it is as follows:

(3) $$U_{O}=A_{o}e^{j\theta_{o}}=(I_{1}-I_{3})+j(I_{2}-I_{4}),$$

This $U_{O}$ represents the low-resolution object wave field. To acquire a high-resolution image, the object field has to be synthetized considering the $\vec{k_{i}}$, and the synthetization is represented as follows:

(4) $$U_{syn}={ℱ}^{-1}\{\sum_{i}ℱ\left\{U_{O,i}\exp\left(-j\vec{k_{i}}\cdot \vec{r}\right)\right\}\}.$$

However, when synthetizing signals, the object wave field has to be clean without distortion. There are many causes that distort the signals. In our system, the distortions are mainly caused by the MLA and the condenser lens. The MLA only consists of spherical lenses which converge the beam to an aberrated point source. The condenser lens, designed to achieve a high numerical aperture (NA) through a simplified optical system, generates various wavefront errors. They are important optical components for generating the directional illumination beam; these make the object wave function too complex to synthetize. The additional problem is that the constant *C* becomes a function depending on *x* and *y* because of the field curvature of the condenser lens, so the phase profile and the direction of the illumination are not predictable.

**Figure 3:**
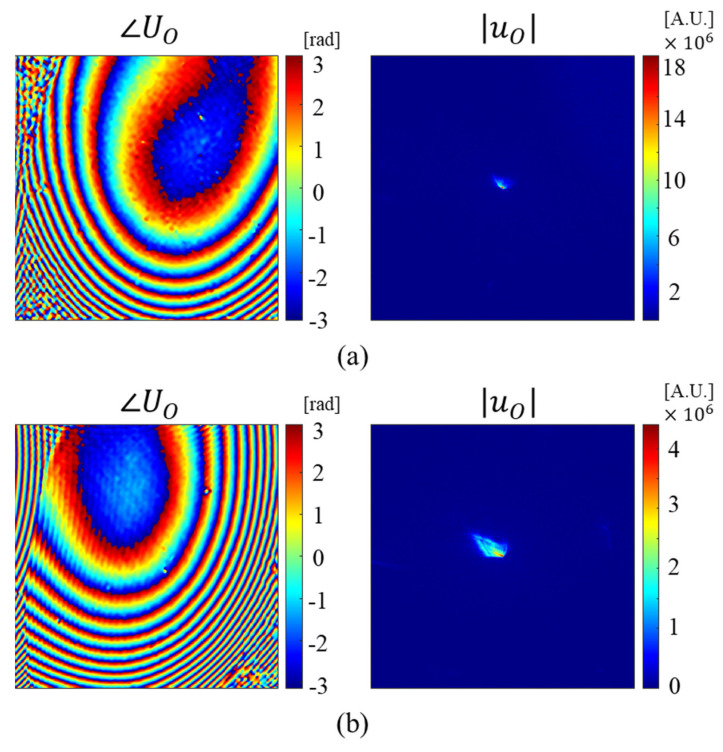
Experimental measurements of intensity phase profiles in focal plane and in Fourier domain. The measurements from the illumination beams are (**a**) $\vec{k_{i}}$ = (122.1, −163.6, 11,808.3) and (**b**) (187.6, −164.6, 11,807.9).

In the dark field region, the beam is not accepted by the objective lens, so it is impossible to directly measure the phase profile of the high-angle illumination beam. Therefore, we introduced a linear grating to redirect the beam into bright field region. Then, the aberration was experimentally measured by the PCCD. Figure 3 shows phase profiles of aberration function and point spread functions of the condenser lens according to the different incident angles. The phase profiles were obtained by the PCCD and the difference between them mainly results from the change of the aberration function of the condenser lens. The point spread function was calculated from the measured phase profiles.

Table 1 represents the normalized Zernike coefficients from the third to the ninth aberration functions for the illumination beam with wave vectors of (122.1, −163.6, 11,808.3) and (187.6, −164.6, 11,807.9), as shown in Figure 3. In this table, the coefficients from the zeroth to the second are not listed. The zeroth term is hard to measure, and represents the piston phase. The variation of the optical path length of the object arm results in the change of the piston phase. We measured the phase profile using the linear grating so that the center of point spread functions were shifted to the origin in the Fourier domain. Therefore, the first and the second terms were not the parameters to be measured, but these two terms needed to be estimated for the synthesis to determine the location of the measurement in Fourier domain. The last terms from the third function to distort the image of the object and the compensation of them are important to improve the quality of the image for each measurement.

## 3. Generation of Training Datasets for DNN Model

A deep neural network (DNN) is an innovative technique that passes input data through hidden layers of a model and extracts inherent features of the data. These processes are learned by the DNN model. The trained model uses these extracted features to effectively predict results. These DNN-based analyses, such as phase profile measurements, are widely used in the field of optics. They involve analyzing interference patterns formed on devices such as CCDs to obtain phase information, or quantifying the distortion level of optical systems. Some researchers quantify the spot of incident beam on the focal plane using a lens instead of an interference pattern. In the case that predicts phase profiles, many DNNs uses a wavefront that is not without loss of the information as input data for the model. However, these approaches sometimes present challenges to certain systems that lose some information of the wavefront. In particular, our SA-DHM encounters a hindrance in obtaining the wavefront diffracted by illumination beams within dark field conditions. To overcome this limitation, we propose a strategy that uses the diffracted pattern from the aberrated illumination as input data with the ResNet architecture. This approach is able to predict Zernike coefficients, which allows the acquisition of phase profiles of the illumination beam in the dark field.

**Figure 4:**
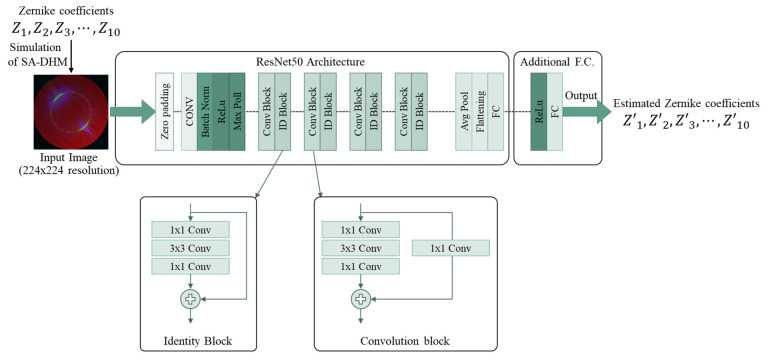
Structure of ResNet50 for Zernike coefficients. The ResNet consists of identity blocks and convolution blocks, and a fully connected layer is added to estimate the ten lowest Zernike coefficients.

Figure 4 shows the basic structure of the ResNet50, which consists mainly of identity blocks and convolution blocks. The identity block is the basic element that forms the ResNet50, and it uses shortcuts and residuals to reduce the effect of the gradient vanishing problem. The convolution block extracts more complex features from an input dataset, and, like the identity block, it concatenates and sums shortcuts with the output of the convolution layer. We used an image with the resolution of 224 × 224 as input data of the model in the ResNet50, as shown in Figure 4. One image was obtained from the simulation of SA-DHM with random Zernike coefficients and it had three channels which correspond to the intensity images in the Fourier domain, a focal plane, and an out-of-focus plane, respectively. A fully connected layer was added to the ResNet50 so that the ten lowest Zernike coefficients were predicted as an output.

**Figure 5:**
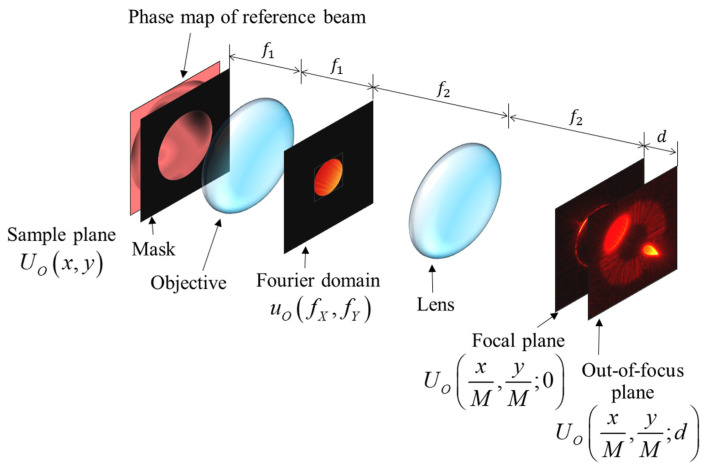
Simplified layout of SA-DHM for generating training and validation data.

To verify that DNNs can be used to extract aberrations of the illumination beam from the diffracted beam, we built a simulation environment, as shown in Figure 5. The parameters used in this simulation were a pixel pitch and the wavelength of 1 micrometer and 0.532 micrometer, respectively. The NA of the simulation was 0.266. The sampling resolution was 2240 × 2240 pixels. A phase map with aberrations was passed through a circular aperture mask which is defines a domain called the Zernike polynomial. Then, the beam was diffracted; here, the diffracted beam can be called a signal. The signal passed through an objective lens with a focal length of *f*_1_, and the beam passing through the objective lens formed a frequency distribution in the Fourier domain away from the objective lens up to *f*_1_. In the Fourier domain, a mask with a 224-pixel diameter was applied to realize the NA of the objective lens, and it meant that the NA of the objective lens 0.0266. The masked signal was Fourier-transformed by the tube lens and imaged onto the CCD plane. As the last procedure, we calculated the intensity map in the out-of-focus region, which is d away from the CCD plane, where *f*_1_, *f*_2_, and *d* are 10, 200, and 10 mm.

We used Zernike coefficients to generate a dataset for DNN model training. We randomly generated Zernike coefficients in a range from 0th to 9th order and generated phase maps based on these coefficients. Here, the range was a set as follows:

(5a) $$Random:\left\{Z_{0}\right\}\to \left[-\pi ,\pi \right],$$

(5b) $$Random:\left\{Z_{1},Z_{2}\right\}\to \left[-1200,1200\right],$$

(5c) $$Random:\left\{Z_{n}\right\}\to \left[-48,\;48\right],$$

where *Random* means that we randomly choose value in the range. *Z*_1_ and *Z*_2_ are related to carrier frequencies, and they are limited to prevent aliasing in the simulation. The coefficients with *n* greater than 3 are randomly chosen in proportion to the carrier frequencies. It is noted that the combinations of all coefficients which are randomly chosen present an arbitrary illumination phase. We generated a total of 100,000 samples. After generation, we input the phase maps into our simulation, then we calculated the results, which is the intensity map on Fourier domain, focal plane, and out-of-focus plane. The three intensity maps were normalized and finally combined as three-channel image files.

**Figure 6:**
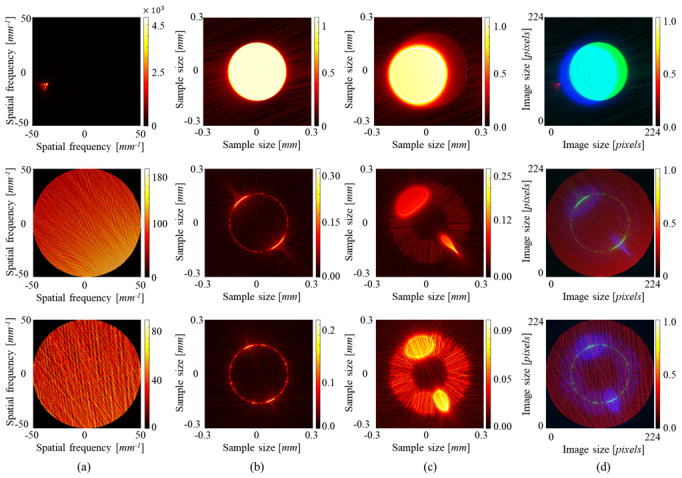
Examples of the training data. The intensity patterns on (**a**) the Fourier domain, (**b**) the focal plane, and (**c**) the out-of-focus plane. (**d**) They are, respectively, occupied in R, G, B channels for training data. The pictures on the first line were numerically generated in the bright field condition and the others were numerically generated in the dark field condition.

Figure 6 shows sample images containing three intensity maps on three channels in one image file. Almost all samples were generated under the dark field condition. The diffraction patterns from the edges of the mask can be observed, as well as the shape of the pattern change depending on the phase profile of the illumination beam. As shown in Figure 6, there was one sample under the bright field condition; the sample shows the filled circle. In the next section, we explain the training results of the model using that sample, and predict the Zernike coefficients.

## 4. Aberration Compensation Using Deep Neural Network

**Figure 7:**
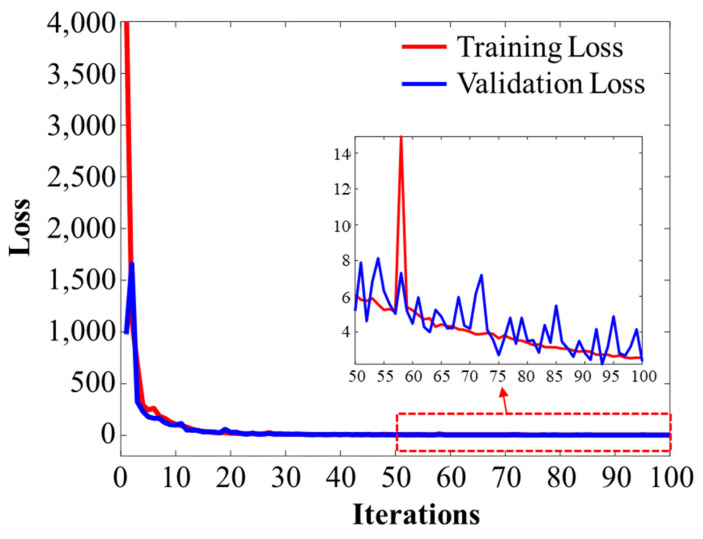
Training history of the loss graph. Red and blue lines represent training loss and validation loss, respectively.

Figure 7 shows the loss function during training of the model with the training and validation data introduced in Section 3. After a total of 100 epochs, the final loss value was about 2.3. Due to the convergence of the validation loss and the training loss, it seems that the training was carried out effectively without overfitting. When we checked the prediction results performed in the middle of the process, it was difficult to predict *Z*_0_, which represents the piston phase, and the final loss value was mainly due to errors in this *Z*_0_ coefficient. The reason for the difficulty in accurately predicting *Z*_0_ is that the piston value represents an overall phase shift that does not significantly affect the intensity maps in both the frequency domain and image domain. Thus, it is difficult for the DNN model to learn any feature about *Z*_0_; therefore, it struggles with prediction.

**Figure 8:**
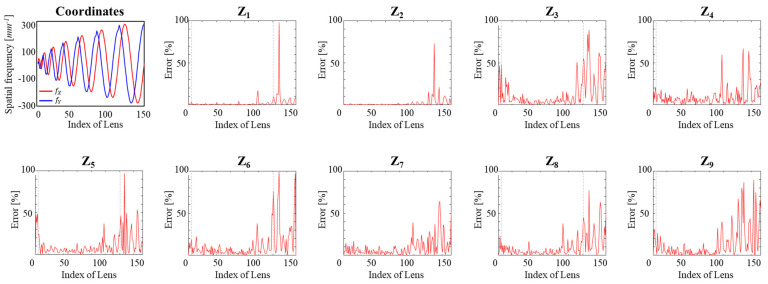
Estimation errors in nine Zernike coefficients of aberration of illumination beams.

Prior to running the SA-DHM simulations, we generated illumination phase profiles using Zernike coefficients related to MLA and aberrations, corresponding to the lens positions *x_i_* and *y_i_*. A total of 153 illumination phase profiles were generated. Here, samples 1 through 5 represent the bright field, and others represent the dark field. Samples 6 through 121 were generated under the range of coefficients that was the same as the range of the training data. On the other hand, samples 122 through 153 were generated using coefficients with a larger range than the training range. This was performed to evaluate how accurately the model predicts results over a larger range. Using the generated illumination phase profiles and a circular aperture in SA-DHM simulations, we generated $I_{n=1,2,3,4}$ on the CCD plane, and calculated *U*_0_ using Equation (3). The calculated *U*_0_ was utilized to generate intensity maps in the frequency domain, the CCD plane, and out-of-focus plane, and these three types of intensity maps were combined into one BMP file. A total of 153 BMP files were generated. We predicted the Zernike coefficients using the trained model from these BMP files, and Figure 8 shows the spatial frequency of the illumination beam according to the lens index and the errors between the actual Zernike coefficients and the coefficients predicted by the trained model. For inputs up to 153, we saw the most error within 50% of the prediction results, and this means that the model has to be used within the training range. These results mean that it seems reasonable to use the predicted result in the range for the SA-DHM.

**Figure 9:**
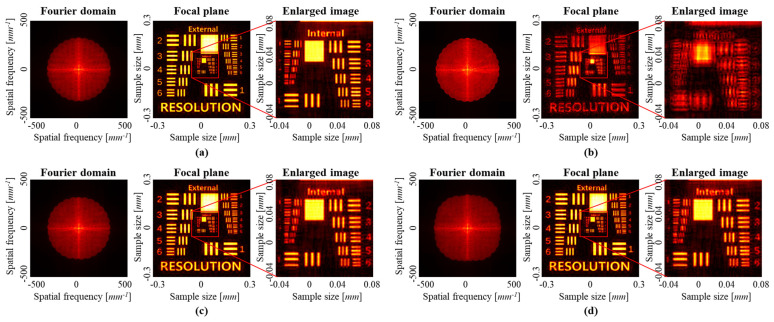
SA-DHM reconstruction results of synthesized intensity profiles in Fourier domain, reconstruction images in focal plane, and their enlarged images. (**a**) Ground truth and (**b**) reconstruction without compensating aberration. Reconstructions with compensating the aberration (**c**) within and (**d**) outside the range of training data.

Figure 9 shows the reconstruction results of an SA-DHM, and the sample was used with an image file based on the USAF resolution target. In this simulation, all *Z*_0_ were used with the ground truth values. Figure 9a shows the reconstruction results using the perfect illumination phase profile, and Figure 9b shows the results using the ground truth *Z*_1_ and *Z*_2_ values without aberration compensation during the reconstruction process. In the simulation results, it is difficult to distinguish high-resolution patterns since the aberrations were not compensated. On the other hand, Figure 9c shows the result of using the predicted Zernike coefficients by using the trained model, with aberration compensation. The result shows the high-resolution image that was reconstructed with a high degree of restoration, which was similar to the actual values. Figure 9d shows the result of restoring the sample with a larger range of coefficients than the training range, i.e., lenses up to the 151st. Although the coefficients predicted from the illumination beam had an error of up to 10%, we can see that a high-resolution restoration of the sample was achieved.

**Figure 10:**
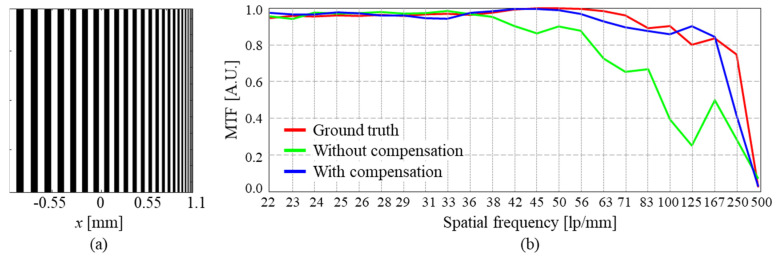
Resolution analysis of the SA-DHM. (**a**) Resolution target. (**b**) MTF chart with and without compensating aberration by using the proposed DNN.

Figure 10 shows the resolution analysis. A linear MTF chart was used as the test target, of which the size increased gradually from 21 lp/mm to 500 lp/mm. It is noteworthy that the maximum resolution of this simulation was 500 lp/mm. The red line is the ground truth. The MTF of the red line is maintained above 0.8 until 167 lp/mm. The green and blue lines represent the MTFs of simulated results before and after compensating aberration, respectively. In the case before compensation, the MTF was maintained high just until 38 lp/mm, but after compensation, the MTF had a very similar tendency to that of the ground truth. In addition, the MTF of the red line decreased rapidly above the certain point of 250 lp/mm. The reason can be interpreted as follows. For example, if the signal is synthesized up to about 60% in the Fourier domain, the maximum resolution becomes around 312.5 lp/mm.

**Figure 11:**
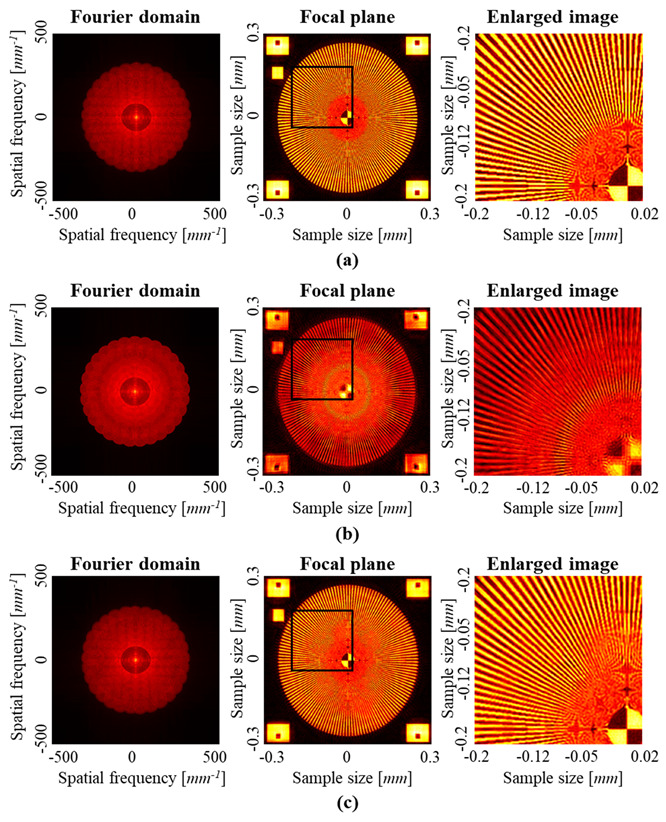
SA-DHM reconstruction results with the spoke resolution chart, and their enlarged images. (**a**) Ground truth and (**b**) reconstruction without compensating aberration. (**c**) Reconstructions with compensating the aberration.

Figure 11 shows additional simulation results. In the simulation, the spoke resolution chart was used as the resolution target, and it had radial lines of which the line width increased far away from the center. Figure 11a shows the reconstruction result by using the ground truth of the Zernike coefficient. Figure 11b,c show the reconstruction results before and after aberration compensation. In the result before compensation, the lines beyond 0.15 mm away from the center are distinguishable. It is worthwhile noting that the lines around the region 0.07 mm away from the center become very sharp. This phenomenon meets the simulation of MTF chart in Figure 10, where there is a peak at 167 lp/mm. In the result after compensation, the region where the lines are distinguishable is a little smaller than that in ground truth. It is also expected from the MTF graph in Figure 10. In addition, the bandwidth of the spectrum in Fourier domain is limited in Figure 11. Therefore, the lines are blurred in the central region with the radius of 0.05 mm. This effect is also found in the MTF graph in Figure 10, where MTFs above 250 lp/mm fall abruptly.

## 5. Discussion

**Figure 12:**
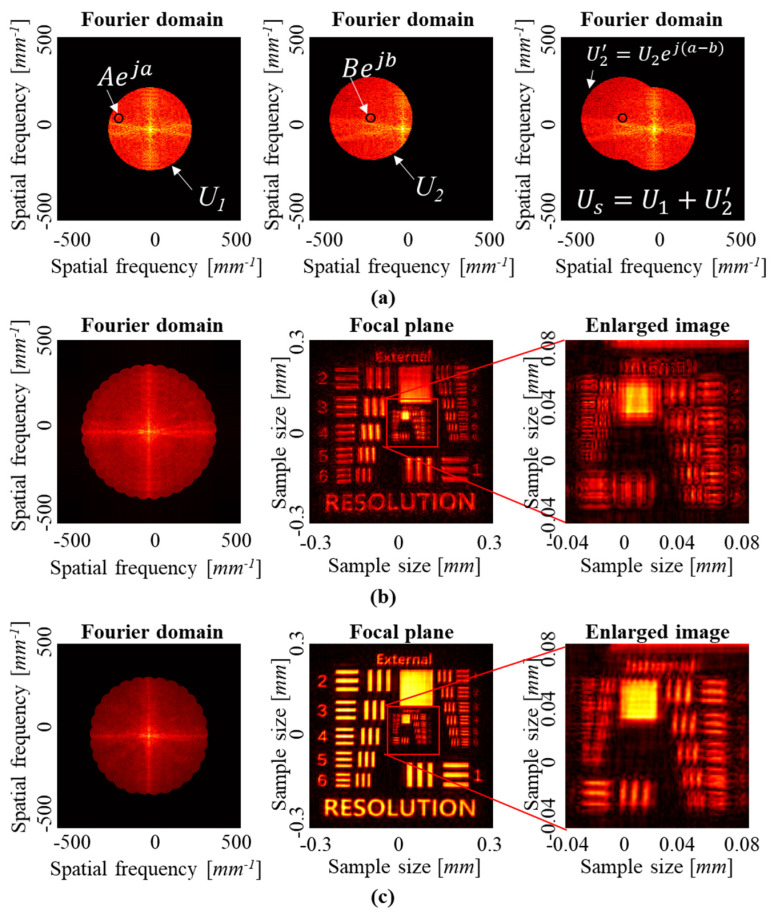
Piston phase matching and numerical reconstruction results. (**a**) Piston phase matching at a specific point in overlapped region. Numerical reconstruction results (**b**) without and (**c**) with piston phase matching.

Aberration compensation in SA-DHM is critical for achieving high-resolution and accurate imaging, because the aberrations degrade the quality of reconstructed images significantly. Our proposed method, which involves training a DNN on Zernike coefficients and diffraction patterns obtained from a circular aperture, aims to address the challenges associated with compensating aberrations beyond the objective lens’ NA. Unfortunately, as explained earlier, the coefficient of *Z*_0_ cannot be predicted by the trained model because the diffraction pattern is not changed by the coefficient. Therefore, considering that *Z*_0_ is unpredictable, we made the phase of Fourier domain of the first wave function at a specific point in the overlapped regions match the piston phase, as shown in Figure 12a. If we perform SA-DHM without matching the piston phases, as shown in Figure 12b, the reconstructed image is severely distorted, and it is difficult to distinguish the original pattern. On the other hand, by compensating for the piston phase, as shown in Figure 12c, we obtain a reconstructed image with more clearly distinguishable patterns.

## 6. Conclusions

In this study, we proposed a method to compensate aberrations in SA-DHM using a DNN. Conventional methods for aberration compensation have the problem of compensating aberrations with high carrier frequency beyond the NA of the objective lens. Our proposed method addressed this problem by training a DNN on Zernike coefficients and diffraction patterns obtained from a circular aperture. Using the estimated Zernike coefficients, the aberration was successfully compensated in the SA-DHM simulation. The reconstruction quality of high-resolution images was dramatically improved when compared to reconstructions without aberration compensation. The ResNet was successfully trained to estimate Zernike coefficients. In addition, the error between the estimation and ground truth remained within reasonable limits even for input values beyond the training range. This indicates the generalizability of the DNN in handling aberrations over a wide range of conditions. Although the estimation of the piston phase coefficient remained challenging, we addressed this issue by adjusting the piston phase in the reconstruction process. Now, we are constructing the SA-DHM and we will attempt to apply the proposed method to our SA-DHM in the near future.

## Figures and Tables

**Table 1:** Examples of normalized Zernike coefficients of aberrations of illumination beams with wavevector $\vec{k_{i}}$.

Zernike Polynomial	Equations	Coefficients	
		(122.1, −163.6, 11,808.3)	(187.6, −164.6, 11,807.9).
0	1	-	-
1	ρsinϕ	-	-
2	ρcosϕ	-	-
3	$\rho^{2}\sin2\phi$	0.5826	10.7284
4	$2\rho^{2}-1$	−4.8872	−11.1697
5	$\rho^{2}\cos2\phi$	−0.8507	−1.1836
6	$\rho^{3}\sin3\phi$	−0.8823	4.3718
7	$\left(3\rho^{3}-2\rho \right)\sin\phi$	1.1083	10.0527
8	$\left(3\rho^{3}-2\rho \right)\cos\phi$	−1.2794	−9.5085
9	$\rho^{3}\cos3\phi$	0.7599	−2.6971

## Data Availability

Data underlying the results presented in this paper are not publicly available at the time of publication, but may be obtained from the authors upon reasonable request.
